# High Affinity: Making Up for Being Male

**DOI:** 10.1371/journal.pbio.0020387

**Published:** 2004-10-05

Because males and females possess different numbers of the two sex chromosomes (for instance, in mammals, XX in females versus XY in males), the potential “dose” of each gene differs. Without some compensating mechanism, female mammals would express twice the quantity of an X-linked gene as males. The same holds true in the fruitfly Drosophila, in which the female carries two X chromosomes, while the male carries only one.

In mammals, dosage compensation is achieved by silencing one of the X's in the female. Drosophila takes the opposite tack, doubling the output from the single male X chromosome. It does so through the creation of “compensasomes,” protein–RNA complexes that bind to the X chromosome and boost gene transcription. One model of compensasome activity has posited a two-step mechanism, in which the complexes form only at 35–40 specific “entry sites” along the X, and then spread out to the surrounding regions. In this issue, Delphine Fagegaltier and Bruce Baker test this model and show that its predictions do not match experimental results.

The compensasome complex includes half a dozen proteins collectively known as MSLs (for “male-specific lethal”), along with two pieces of RNA, *roX1* and *roX2*. Fagegaltier and Baker reasoned that, according to the entry-site model, if a piece of the X not containing one of the entry sites was transposed to an autosome (non-sex chromosome), it should be unable to recruit MSLs and therefore be unable to form compensasomes. To test this prediction, they used autosomes into which various pieces of the X had been transposed. Contrary to prediction, they found that even the smallest pieces could recruit MSLs, whether or not they contained entry sites. Furthermore, the pattern of MSL binding was exactly the same as if the fragment of the X was still on its native chromosome, suggesting that each of the hundreds of sites at which compensasomes are found function autonomously to recruit them.

**Figure:**
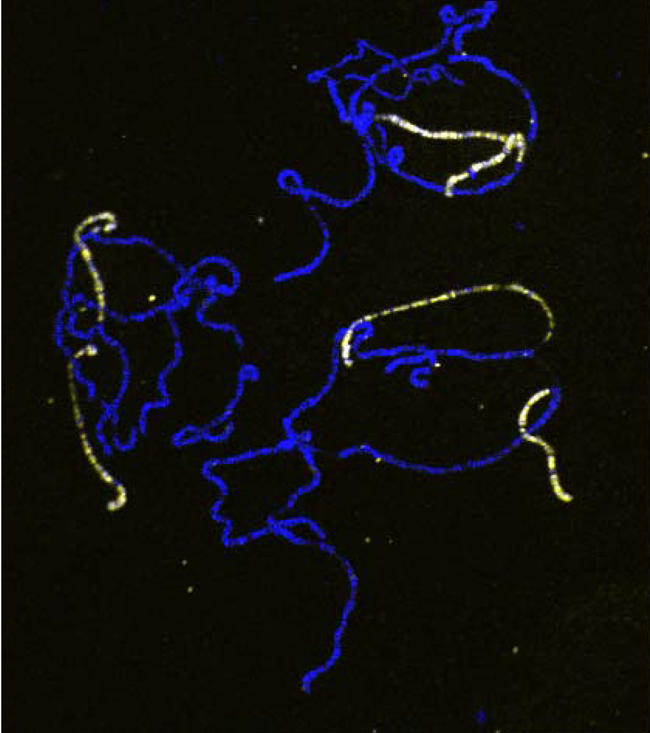
Compensasomes do not spread from the X chromosome onto autosomal material translocated onto the X

Another prediction of the entry-site model is that compensasomes should spread out from the entry site, along the chromosome. And here again, the model does not hold up—Fagegaltier and Baker found that even when entry sites from the X chromosome are put close to an autosomal region, compensasomes never spread from the X onto these regions. These results suggest that spreading is not an innate function of the compensasome, and further strengthens the case for autonomous recruitment all along the X. In place of the two-step “entry site plus spreading” model, the authors propose a model based on differential affinity for compensasome components. They suggest that the 35–40 “entry sites” are simply high-affinity sites that recruit MSLs first, based on intrinsic differences that allow them to bind and hold MSLs more strongly than other sites. Once these sites are occupied, additional compensasome components can bind to lower-affinity sites. This mechanism can account for observed compensasome activity without the restriction to a limited number of entry sites and the requirement for spreading.

Fagegaltier and Baker note that while compensasome spreading does not normally occur during dosage compensation on the X chromosome, it has nonetheless been documented for some *roX* transgenes. They propose that the additional binding observed specifically around *roX* transgenes results from a mass action of compensasomes, as *roX* transgenes would act as assembly sites for compensasomes, just as ribosomal RNA genes do for ribosomes. Once formed, compensasomes may bind locally to other neighboring sites.

While the details of dosage compensation and the dosage compensation complexes now clearly differ between mammals and flies, there are broad similarities, including the widespread modification of chromatin structure and the use of RNA components in the compensation machinery. A deeper understanding of the process in flies may help shed light on the details of compensation in other organisms as well.

